# Identification and functional analysis of circulating small extracellular vesicle lncRNA signatures in children with fulminant myocarditis

**DOI:** 10.1111/jcmm.18034

**Published:** 2023-11-09

**Authors:** Mengjie Ma, Siyu Chen, Xinyue Zhang, Rulin Yang, Li Zhang, Kaiyin Guo, Jing Wang, Hailin Jia, Yingnan You, Bo Han

**Affiliations:** ^1^ Department of Pediatrics, Shandong Provincial Hospital Shandong University Jinan Shandong China; ^2^ Department of Pediatrics The Second Affiliated Hospital of Shandong First Medical University Taian Shandong China; ^3^ Department of Pediatrics Shandong Provincial Hospital Affiliated to Shandong First Medical University Jinan Shandong China

**Keywords:** fulminant myocarditis (FM), functional prediction, immune, long non‐coding RNA (lncRNA), small extracellular vesicles (sEVs)

## Abstract

Fulminant myocarditis (FM) is the most serious type of myocarditis. However, the molecular mechanism underlying the pathogenesis of FM has not been fully elucidated. Small extracellular vesicles (sEVs) play important roles in many diseases, but any potential role in paediatric FM has not been reported. Here, the differential signatures of lncRNAs in plasma sEVs were studied in FM children and healthy children using transcriptome sequencing followed by functional analysis. Then immune‐related lncRNAs were screened to study their role in immune mechanisms, the levels and clinical relevance of core immune‐related lncRNAs were verified by qRT‐PCR in a large sample size. Sixty‐eight lncRNAs had increased levels of plasma sEVs in children with FM and 11 had decreased levels. Functional analysis showed that the sEVs‐lncRNAs with different levels were mainly related to immunity, apoptosis and protein efflux. Seventeen core immune‐related sEVs‐lncRNAs were screened, functional enrichment analysis showed that these lncRNAs were closely related to immune activation, immune cell migration and cytokine pathway signal transduction. The results of the study show that sEVs‐lncRNAs may play an important role in the pathogenesis of fulminant myocarditis in children, especially in the mechanism of immune regulation.

## 1 INTRODUCTION

Myocarditis is an inflammation of the myocardial tissue caused by infection, immunity, poisoning or other factors. It is also one of the most common cardiovascular diseases in children.^1^ Fulminant myocarditis (FM) is the most serious type of myocarditis, with a rapid onset and severe illness progression, which can lead to heart failure, severe hemodynamic compromise, cardiogenic shock, multi‐organ failure and death.^2^ Previous work has shown that immune response dysregulation, inflammatory factor storms and direct pathogenic microorganism invasion of cardiomyocytes are all important components of the pathogenesis of FM,^3^, ^4^, ^5^ but the specific molecular mechanisms have not been fully elucidated.

Non‐coding RNAs longer than 200 nucleotides are referred to as long non‐coding RNAs (lncRNAs).^6^ LncRNAs have complex secondary and tertiary structures, which enables them to have much richer biological functions.^7^, ^8^ At the epigenetic and transcriptional levels, lncRNA can activate, serve as a decoy, guide and/or scaffold of effector proteins.^9^ At the post‐transcriptional level, lncRNAs can serve as editing regulators of RNA, miRNA blockers, and regulators of RNA shearing and decomposition.^10^

The development of high‐throughput sequencing technology has vastly enhanced our studying of lncRNA. The role of lncRNA in cardiovascular diseases has attracted widespread attention.^11^, ^12^, ^13^, ^14^ Prior studies have found that abnormal lncRNA expression is related to various pathophysiological processes, including proliferation and differentiation of cardiomyocytes, cardiac remodelling, cardiac stress and immune regulation.^15^, ^16^, ^17^ In addition to regulating intracellular functions, lncRNAs can also be released into the extracellular environment and act on other cells or serve as markers of disease processes.^18^ Circulating lncRNAs are closely related to small extracellular vesicles (sEVs).^19^ Researchers have found that lncRNAs carried by sEVs can play an important role in cardiovascular diseases. For example, Lei Wang et al. found that lncRNA AK139128 from the hypoxic cardiomyocyte exosome could regulate the apoptosis and cellular activity of fibroblasts.^20^ Qing Mao et al. found that sEV‐lncRNA KLF3‐AS1 derived from mesenchymal stem cells could regulate myocardial apoptosis via the miR‐138‐5p/Sirt1 axis, meaning they have an important role in myocardial infarction.^21^

Although the benefit of studying EV‐lncRNAs in cardiovascular disease has been established, there have been no studies about it in children with myocarditis. In this study, we used second‐generation sequencing to characterize lncRNAs in plasma sEVs of children with fulminant myocarditis and in healthy children. Then performed functional enrichment analysis of lncRNAs with different levels. After that, we screened for sEVs‐lncRNAs that played important roles in immune regulation and made function analysis. The data presented here may help generate new perspectives on the pathophysiology of FM and ultimately support the search for new therapeutic targets.

## 2 MATERIALS AND METHODS

### 2.1 Patient information and plasma sample collection

We included 15 children diagnosed with FM who were treated in the Pediatrics of Shandong Provincial Hospital between January 2021 and May 2022, as well as 15 healthy volunteers of similar ages and sexes to serve as a control group. Informed consent was signed by the parents of each subject (age < 18). This study strictly adhered to the Declaration of Helsinki and was approved by the Shandong Provincial Hospital Ethics Committee. All patients with FM were clinically diagnosed according to the 2013 position statement of the European Society of Cardiology,^22^ the Expert Consensus Standard of the Chinese Society of Cardiology^23^ and the Scientific Statement on the Diagnosis and Treatment of Childhood myocarditis from the American Heart Association.^1^

Peripheral blood samples (8 mL) were collected using an EDTA tube through a routine venipuncture procedure as fasting blood tests. After being centrifuged at 4°C for 10 min at 1900 *g*, the supernatant was removed, and the samples were centrifuged at 1600 *g* and 4°C for 10 min. The supernatant was stored at −80°C until use.

### 2.2 sEV separation

A total of 4 mL of plasma was collected from each participant, and sEVs were extracted by ultracentrifugation. After being thawed at 37°C, the plasma samples were centrifuged at 3000× *g* for 15 min, the supernatants were diluted with seven times the volume of PBS. After centrifugation at 13000× *g* for 30 min. The supernatants were filtered into the overspeed centrifugal tube using the P50AT2‐986 rotor (CP100NX; Hitachi, Brea, CA, USA), and were then centrifuged at high speed at 150,000× *g* at 4°C for 4 h. The precipitates were resuspended in PBS and centrifuged again at 150,000× *g* 4°C for 2 h. The sEV‐enriched precipitates were then resuspended in 200‐μL PBS.^24^

### 2.3 Nanoparticle tracking analysis (NTA)

Particle size and the quantity of isolated particles were measured using ZetaViewPMX120 (Particle MetrixGmbH, Ning Amansi, USA, Germany). The ideal measured concentration was the ideal particle concentration determined by pre‐testing each frame value (140–200 particles/frame), and was completed for each measurement by scanning 11 cell sites for two cycles. After capture, NTA software (ZetaView 8.05.05SP2) was used to analyse the particle motion. The specific analysis parameters were 1000, the minimum particle size was five and the minimum particle brightness was 10.

### 2.4 Transmission electron microscope (TEM)

10 μL of sEV suspension was added to a 200 mesh carbon film copper net and incubated at room temperature for 20 min. The copper mesh was fixed on 50‐μL 1% glutaraldehyde liquid drops for 5 min. After ddH_2_O_2_ washing, the uranium dioxyacetate solution and the ratio of methyl cellulose to uranium dioxyacetate were successively used for 5 min. The excess liquid was absorbed and dried under an incandescent lamp for 5 min. We then observed and photographed the copper mesh under the TEM (HT‐7700, Hitachi, Japan).

### 2.5 Western blot analysis

The sEV suspension was denatured with 4× sodium dodecyl sulfonate (SDS) buffer and analysed using western blotting (10% SDS‐polyacrylamide gel electrophoresis; 30 μg protein/well), with monoclonal rabbit antibodies CD63 (1:3000 Abcam Cat#ab134045, USA), TSG101(1:3000 Abcam Cat#ab125011, USA), CD81 (1:3000 Abcam Cat#ab109201, USA) and calnexin (1:3000 Abcam Cat#ab133615, USA). These proteins were analysed automatically on the Tanon4600 automated chemiluminescence image analysis system.

### 2.6 RNA isolation and quality control

Total RNA from the sEVs was extracted using the miRNeasy® Mini kit (Qiagen, cat217204). RNA concentration, purity and integrity were assessed using the RNA 6000 Nano detection kit from the Agilent Bioanalyzer 2100 system (Agilent Technologies, CA, USA).

### 2.7 Library establishment and sequencing

Eligible RNA samples were constructed using an ultra‐sensitive trace sample chain specific kit for sEV‐RNA. The constructed libraries were tested for concentration using a Qubit® 2.0 Fluorometer and by size with Agilent2100. Qualified libraries were prepared for Illumina sequencing with the PE150 sequencing strategy. After obtaining raw data, the data were filtered, sequenced and processed with low‐quality reads. Next, after evaluating sequencing quality, clean data were obtained with a repeated inspection. The HISAT2 software was used to conduct genome mapping analysis of the pre‐processed sequencing sequences, and comparisons were made with the reference genome GRCh38. Known mRNA and lncRNA were quantitatively analysed. Data analysis was conducted by Agilent Technologies at Sinotech Genomics Corporation.

### 2.8 lncRNA quantitative and differential analysis

We applied cuffcompare in cufflinks (version:2.1.1) to compare the annotation information we obtained from mapping to the reference annotation (in the NONCODE and Ensembl databases), and found new transcripts that did not match the known annotation genes. lncRNA prediction was performed by extracting transcripts which was a transfrag falling entirely within a reference intron, unknown, intergenic transcript or exonic overlap with reference on the opposite strand. We applied Stringtie (version:1.3.0) to the predicted novel lncRNA and NONCODE databases (http://www.noncode.org/), and to the known lncRNAs in the Ensembl database, for quantification of expression. The TMM algorithm in the edgeR software was used for normalization, and *p*‐values were calculated according to the hypothesis testing model. Finally, we corrected *p*‐values to account for multiple testing and obtained FDR values (*q*‐values). Differential expression multiples (fold change) were calculated using FPKM values. mRNAs and lncRNAs with *p* < 0.05 and log2‐fold changes >2 were considered to be differentially expressed between groups. We drew volcano maps and heat maps for mRNAs and lncRNAs with different levels.

### 2.9 Target gene prediction and GO/KEGG pathway enrichment analysis

Interactions between lncRNA and mRNA are divided into cis and trans categories.^25^ Cis target gene prediction looks for mRNA located in the upper and lower parts of lncRNA within 10 kb of the target gene. LASTAL prediction model based on base complementary pairing principle was used to predict trans target gene.^26^ Blast alignment was used to obtain mRNA that was complementary with lncRNA. Then, RNAplex software was used to calculate the thermodynamic parameters of lncRNA that were complementary with mRNA, and sequences with e's < =30 were selected.

To better understand the biological functions that these sEVs‐lncRNAs with different levels may perform in cells or signalling pathways, we examined the functional enrichments of target genes corresponding to these sEVs‐lncRNAs in the Gene Ontology (GO) and the Kyoto Encyclopedia of Genes and Genomes (KEGG) databases. We used the ‘clusterProfiler’ package in the R software package for Fisher precise tests to construct gene annotations.

### 2.10 Screening of immune‐related genes

We obtained a list of immune‐related genes from the IMMPORT (http://www.immport.Org) database, which covers a total of 17 immune classes based on different molecular functions.^27^ Venn diagram analysis was used to identify immune‐related target genes of sEVs‐lncRNAs with different levels.

### 2.11 PPI network construction and core immune‐related sEV‐lncRNA screening

String11.5 (https://string‐db.org) is a database that searches for interactions between known and predicted proteins.^28^ We used String11.5 for PPI network analysis of immune‐related target genes. PPI networks were analysed by uploading mRNA and selecting the correct species (*Homo sapiens*), hiding the nodes where the network disconnects, and using a confidence level > 0.4. The networks were visualized using Cytoscape 3.9.1. The cytoHubba plug‐in in Cytoscape was used to find top 20 hub genes in six ways (MCC, MNC, Betweenness, Degree, EPC and Stress), and we used the intersections to screen core genes. Then the core immune‐related sEVs‐lncRNAs were deduced according to immune‐related target genes.

### 2.12 qPCR quantified the levels of core immune‐related sEVs‐lncRNAs

Total RNA from sEVs was extracted using the miRNeasy Mini kit (Qiagen, cat 217,184). cDNA Synthesis was performed using the PrimeScript II 1st Strand cDNA Synthesis Kit (TaKaRa, cat 6210A China). Using the TB Green Premix Ex Taq II (Takara cat RR820A, China), LncRNA was quantified with RT‐qPCR in the LightCycler480 system (Roche Diagnostics, Switzerland). The primer sequence, as shown in the Table S1, was synthesized by DIAUPBIOTHECH Co., LTD. (China). The relative level was determined by 2^−△△Ct^.

### 2.13 Statistical analysis

SPSS26.0 (SPSS, IL, USA) and GraphPadPrism9.0 (GraphPad software, CA, USA) were used for data analysis. Data normality was tested using the Shapiro–Wilk (S–W) test, for data conforming to normal distributions, the mean ± standard deviation was reported, and Student's *t* tests were used to compare data between the two groups, for data that do not conform to normal distribution, Mann–Whitney *U* test was performed to compare the data between the two groups. The Pearson or Spearman rank correlation was used to analyse the correlation between the two variables. The ROC curve analysis was used to assess the utility of candidate lncRNA as diagnostic biomarker to distinguish paediatric FM patients from NCs. All tests were performed three or more times. All tests were two tailed with multiple comparative controls for false discovery rate (FDR). *p* values <0.05 were considered statistically significant.

## 3 RESULTS

### 3.1 Characteristics of plasma‐derived sEVs‐enriched components in children with FM

**FIGURE 1:**
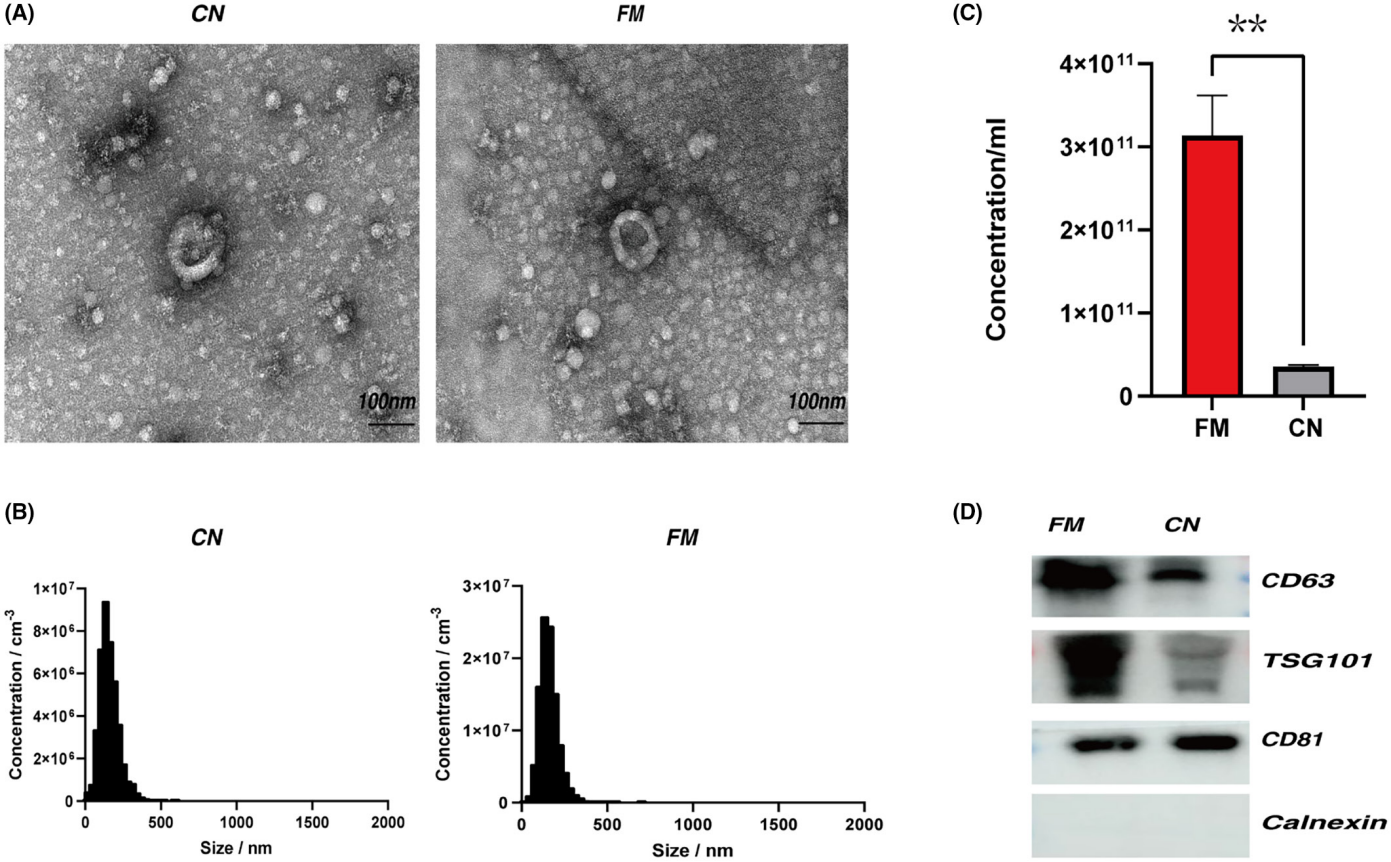
Isolated sEVs‐enriched fractions from participants' plasma. (A) TEM images showed that sEVs were bowl‐shaped capsules without the nucleus, the left panel showed sEVs in the normal control group and the right panel shows sEVs in the FM group. (B) NTA results suggested that sEVs enriched from plasma were about 75‐200 nm in diameter. (C) Comparison of plasma sEVs concentration between FM group and NC group (*p* = 0.00460). (D) sEVs markers CD63, TSG101 and CD81 were all detected in the sEVs‐enriched fractions isolated from the plasma, and the negative marker of sEVs, calnexin, was absent in our isolated sEVs‐enriched fraction samples.

In this study, we recruited 15 children with FM and 15 healthy children as controls (NC). The basic information of each participant and the clinical courses of children with FM are shown in Tables S2 and S3. sEVs were extracted from participants' plasma, separated using ultracentrifugation, and morphologically analysed with transmission electron microscopy (TEM). The size and particle number of sEVs were revealed by nanoparticle tracking analysis (NTA). The results showed that enriched sEVs were elliptical or single‐sided concave saucers in shape and were smaller than 200 nm (Figure 1A,B). Our results also showed that the plasma sEVs concentration was significantly higher in the FM group than in the control group (Figure 1C). Western blot analysis showed enrichment of sEVs markers CD81, TSG101 and CD63 in sEVs‐enriched components (Figure 1D), but did not detect the negative marker of sEVs, calnexin (Figure 1D). These data all indicate that the isolated components we found were mainly composed of sEVs. In addition, through the quantitative analysis of protein by western blot, we found that the expression levels of sEVs markers CD63 and TSG101 in children with FM were significantly higher than those in healthy children, while the expression levels of CD81 were not different between the two groups, this also confirmed the existence of different EV subgroups in plasma of different populations.^29^

### 3.2 Identification of sEVs‐mRNA and sEVs‐lncRNA signatures in the FM and NC groups

**FIGURE 2:**
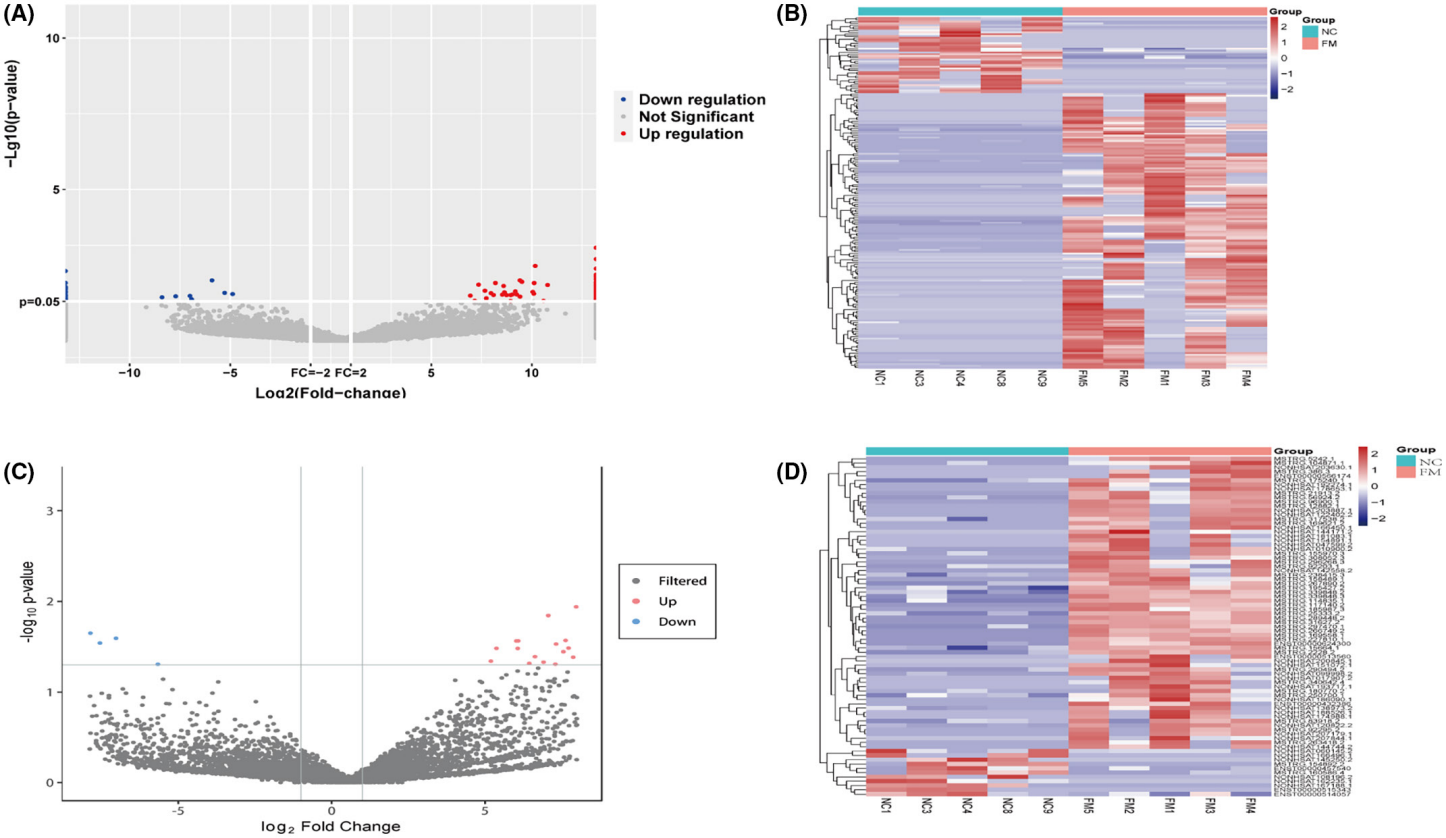
Identification of sEVs‐mRNA and sEVs‐lncRNA signatures between fulminant myocarditis and normal control. (A) Volcano plot showing differentially expressed sEVs‐mRNAs with various *p*‐values and fold changes. Horizontal line, *p*‐value = 0.05 (−log10 scaled); vertical line, fold change = 2 (log2 scaled). Red points, increased sEVs‐mRNAs; blue points, decreased sEVs‐mRNAs. (B) Hierarchical cluster analysis of sEVs‐mRNAs with altered levels (*p* < 0.05, fold change >2) between the two groups. Red strip, the level of mRNAs relative increased in the sEVs; blue strip, the level of mRNAs relative decreased; white strip, no change in gene content. Colour brightness reflects the degree of content increase or decrease. (C) Volcano plot of sEVs‐lncRNAs with different levels. (D) Hierarchical cluster analysis of sEVs‐lncRNAs.

To gain insight into the transcriptional dynamics of extracellular vesicles, we isolated sEVs from the plasma of selected FM children (*n* = 5) and healthy control children (*n* = 5) using ultracentrifugation. Total RNA was extracted from plasma sEVs of the two groups, and RNA sequencing was performed using the Illumina NovaSeq platform. Sequencing results showed that 48,210 transcripts were detected. Of these transcripts, 23,813 were mRNAs and 24,397 were lncRNAs (21,764 known lncRNAs and 2633 presumed new lncRNAs). Compared with the NC group, 194 sEVs‐mRNAs showed more than twofold change and *p* < 0.05 (the level of 152 transcripts were increased and 42 were decreased). SEVs‐mRNAs with different levels are shown in volcano plots (Figure 2A) and heat maps (Figure 2B).

Similar analyses were conducted for sEVs‐lncRNAs. Comparing the FM group and the NC group, we found that 79 lncRNAs transcripts showed statistically significant changes. Sixty‐eight of these transcripts were increased, and 11 were decreased. These different sEVs‐lncRNAs are shown in volcano maps (Figure 2C) and heat maps (Figure 2D).

### 3.3 Functional annotation of sEVs‐mRNAs and sEVs‐lncRNAs with different levels

**FIGURE 3:**
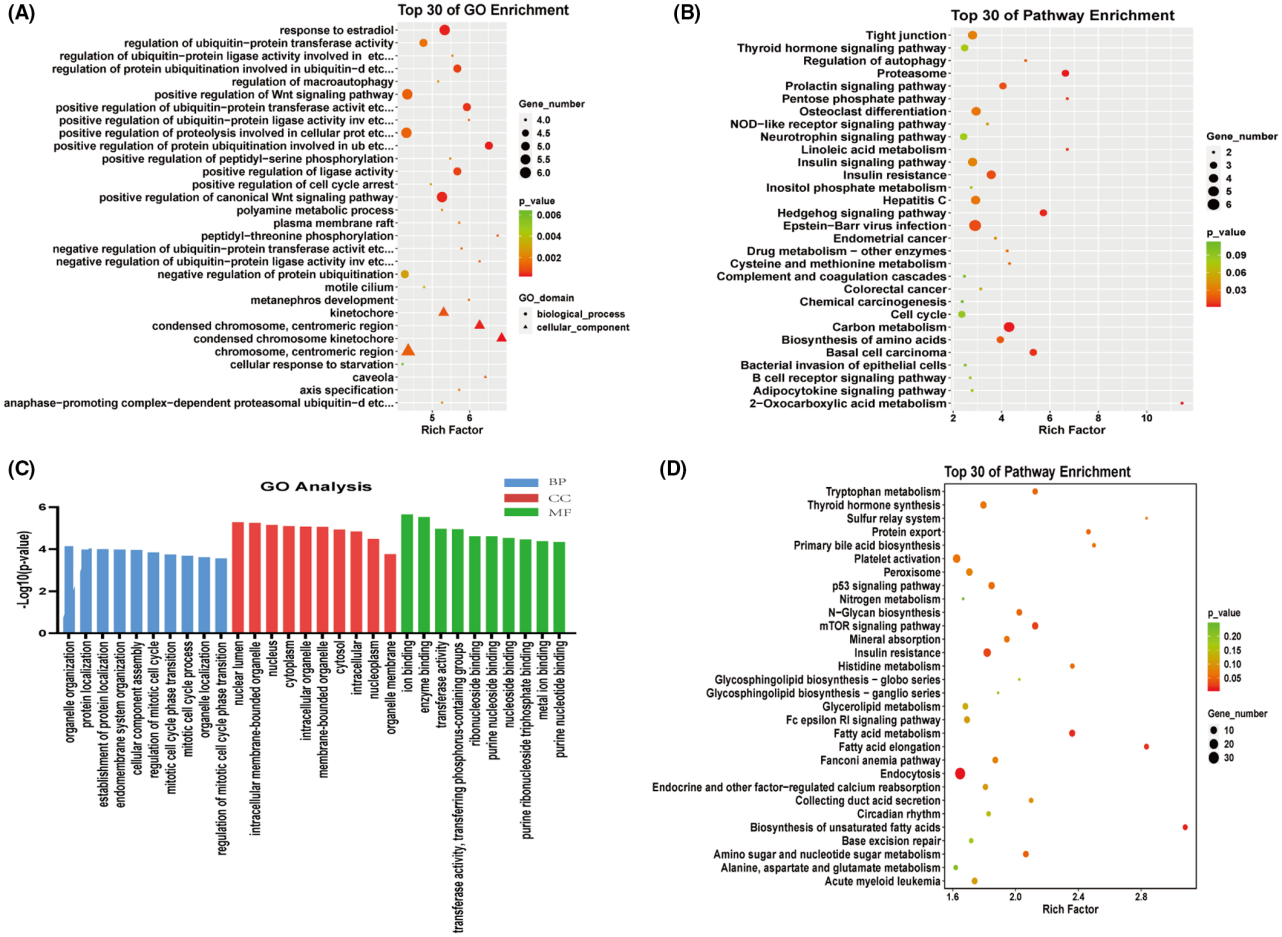
Gene Ontology and Kyoto Encyclopedia of Genes and Genomes pathway analyses of dysregulated sEVs‐mRNAs and sEVs‐lncRNAs in fulminant myocarditis. (A) Top 30 Gene ontology of dysregulated sEVs‐mRNAs. (B) Top 30 Kyoto Encyclopedia of Genes and Genomes (KEGG) pathways of dysregulated sEVs‐mRNAs. (C) Ten most enriched gene ontology terms in biological process, cellular component and molecular function for sEVs‐lncRNAs with different levels. (D) Top 30 Kyoto Encyclopedia of Genes and Genomes pathways of dysregulated sEVs‐lncRNAs. In GO enrichment analysis, the rich factor = (number of differential genes in GO entries/number of all differential genes)/(total number of genes in GO entries in the database/total number of genes in the database); in KEGG enrichment analysis, rich factor = (Number of differential genes involved in the KEGG pathway/total number of differential genes)/(total number of genes in the KEGG pathway in the database/total number of genes in the database).

GO and KEGG pathway analysis was used to gain insight into the biological characteristics of sEVs‐mRNAs with different levels. The top 30 enriched GO terms are shown in Figure 3A.

In terms of biological process, the sEVs‐mRNAs with different levels were most significantly enriched for peptidyl‐threonine phosphorylation (rich factor = 6.75, *p* = 8.70 × 10^−4^), positive regulation of protein ubiquitination involved in ubiquitin‐dependent protein catabolic process (rich factor = 6.51, *p* = 3.48 × 10^−4^) and negative regulation of ubiquitin–protein ligase activity involved in mitotic cell cycle (rich factor = 6.26, *p* = 1.20 × 10^−3^). For the cellular component, the most significant enrichment item was condensed chromosome kinetochores (rich factor = 6.85, *p* = 2.67 × 10^−4^). In the top 30 GO terms, there were no terms under the molecular function category.

KEGG pathway analysis results showed that sEVs‐mRNAs with different levels differentially involved the proteasome pathway (*p* = 0.0027), the Hedgehog signalling pathway (*p* = 0.0046), the Epstein–Barr virus infection pathway (*p* = 0.0132), the signalling pathway that regulates autophagy (*p* = 0.0170) and the NOD‐like receptor signalling pathway (*p* = 0.0461; Figure 3B).

Interactions between lncRNA and mRNA can be divided into cis and trans effects. A total of 1394 target genes were predicted for the 79 sEVs‐lncRNAs with different levels. Of these genes, 1386 were trans target genes, 24 were cis target genes and 2 were both trans and cis target genes.

We used the GO and KEGG pathways to analyse the potential function of these sEVs‐lncRNAs‐related target genes. The GO terms are shown in Figure 3C. For the biological process, the most significant enrichment items were organelle organization (*p* = 6.58 × 10^−5^), protein localization (*p* = 9.37 × 10^−5^) and establishment of protein localization (*p* = 1.00 × 10^−4^). For the cellular component, the most significant enrichment items were related to nuclear lumens (*p* = 5.28 × 10^−6^) and intracellular membrane‐bounded organelles (*p* = 5.66 × 10^−6^). For molecular function, the most significant enrichment items were ion binding (*p* = 2.22 × 10^−6^), enzyme binding (*p* = 2.99 × 10^−6^) and transferase activity (*p* = 1.08 × 10^−5^).

KEGG results showed that genes related to sEVs‐lncRNAs with different levels were mainly involved in endocytosis (*p* = 0.0095), mTOR signalling pathways (*p* = 0.0245) and protein export (*p* = 0.050; Figure 3D).

### 3.4 Screening and functional analysis of immune‐related target genes

**FIGURE 4:**
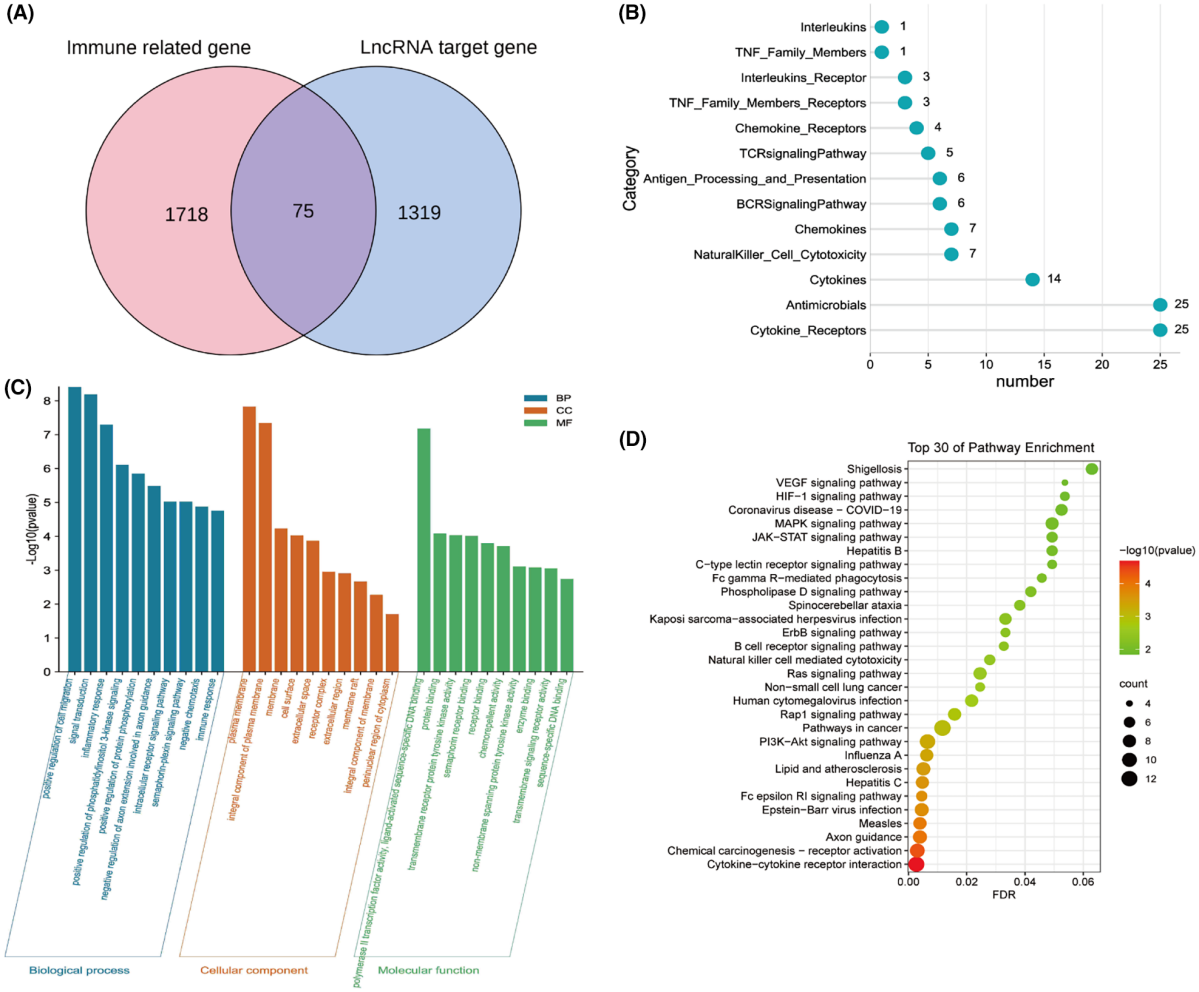
Screening and functional analysis of immune‐related target genes. (A) Venn diagram analysis, to identify the relationship between immune‐related genes and lncRNA target genes. (B) The distribution of identified immune‐related target genes across immune categories. (C) Ten most enriched gene ontology terms in biological process, cellular component and molecular function for immune‐related target genes. (D) Top 30 KEGG pathways of immune‐related target genes.

Previous studies have shown that immune dysregulation is important in the pathogenesis of myocarditis. In order to explore the function of sEVs‐lncRNAs with different levels in the immune regulation of myocarditis, we further screened immune‐related target genes from the predicted target genes and conducted functional enrichment analysis. The IMMPORT (http://www.immport.Org) database is an open access immune‐related gene database that has collected 1793 immune‐related genes from more than 300 studies covering 17 immunity classes. We found 75 immune‐related target genes using Venn diagram analysis (Figure 4A). The distribution of immune‐related target genes in 17 immune‐related categories is shown in Figure 4B. Immune‐related target genes were mainly concentrated in the antimicrobials and cytokine receptors categories.

We used the DAVID (https://david.ncifcrf.gov) database to make an enrichment analysis of the 75 immune‐related target genes through the GO and KEGG pathways (Figure 4C,D). GO analysis showed that, in terms of biological processes, the most significant enrichment terms were related to positive regulation of cell migration (*p* = 3.90 × 10^−9^), signal transduction (*p* = 6.45 × 10^−9^) and inflammatory responses (*p* = 5.05 × 10^−8^). For cellular components, the most significant enrichment terms were plasma membrane (*p* = 1.47 × 10^−8^) and integral component of the plasma membrane (*p* = 4.50 × 10^−8^). For molecular functions, the most significant enrichment terms were polymerase II transcription factor activity, ligand‐activated sequence‐specific DNA binding (*p* = 6.58 × 10^−8^), protein binding (*p* = 8.23 × 10^−5^) and transmembrane receptor protein tyrosine kinase activity (*p* = 9.25 × 10^−5^). KEGG pathway analysis showed that immune‐related target genes were mainly related to cytokine–cytokine receptor interactions (*p* = 2.07 × 10^−5^), Fc epsilon RI signalling pathways (*p* = 2.08 × 10^−4^), PI3K‐Akt signalling pathways (*p* = 4.99 × 10^−4^), Rap1 signalling pathways (*p* = 0.001) and Ras signalling pathways (*p* = 0.003).

### 3.5 Screening of immune‐related core genes

**FIGURE 5:**
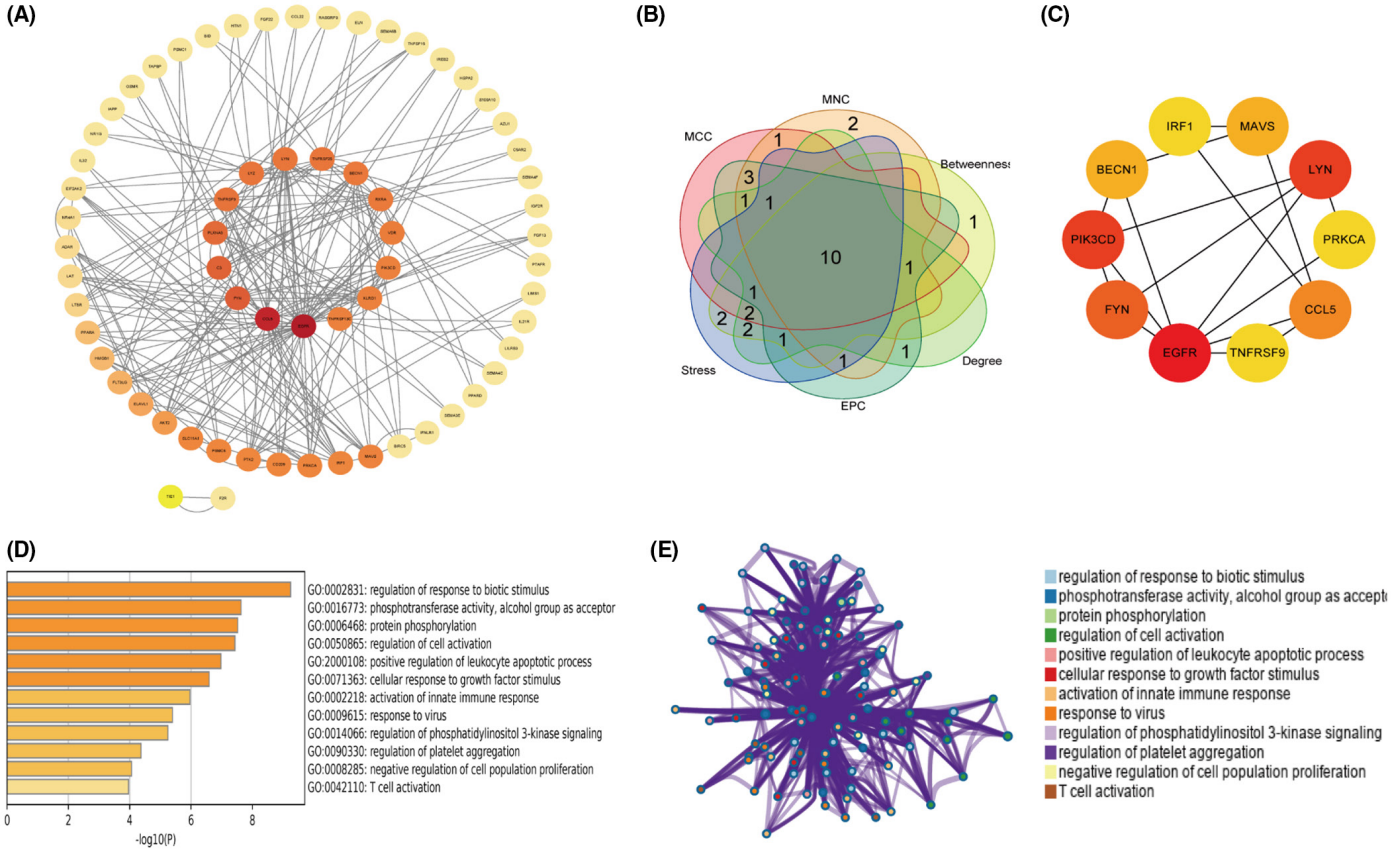
Identification and functional characterization of hub immune‐related target genes. (A) The STRING database is used to construct the PPI network of immune‐related target genes, with 75 nodes and 105 edges. (B) The hub genes of top 20 were screened using six topological methods of the cytoHubba plug‐in in Cytoscape. There were 10 genes in the six methods. (C) The interaction diagram between the ten hub genes.(D) GO enrichment analysis of the biological process of the hub immune‐related target genes. (E) Interaction networks of the enriched biological terms for the hub immune‐related target genes.

To further identify immune‐related target genes that may play key roles in myocarditis, we used STRING to analyse the protein–protein interaction (PPI) network of 75 immune‐related target genes, and visualized the network using Cytoscape 3.9.1. The results were shown in Figure 5A. The PPI network consists of 75 nodes and 109 edges. The top 20 hub genes were screened using six topological methods in the cytoHubba plug‐in in Cytoscape. Ten hub genes were identified using the six methods (Figure 5B), including EGFR, LYN, FYN, PIK3CD, CCL5, PRKCA, IRF1, MAVS, BECN1 and TNFRSF9, and their interactions are shown in Figure 5C. GO functional enrichment analysis of hub genes was performed using the Metascape (https://metascape.org) database (Figure 5D). We found that core genes were mainly enriched for responses to biotic stimulus, regulation of cell activation, positive regulation of leukocyte apoptotic processes and activation of innate immune responses. The interaction network of these biological terms is shown in Figure 5E.

### 3.6 Screening of core immune‐related sEVs‐lncRNAs

Using the hub target genes screened above, we found 17 corresponding lncRNAs, two of which were decreased and 15 of which were increased (Table 1). The gene EGFR was the cis target gene of MSTRG.296268.3, and FYN was both the cis and trans target gene of NONHSAT207844.1. The other 15 lncRNAs with different levels were all trans regulators of their target genes.

**TABLE 1:** Detailed information about the 17 selected sEVs‐lncRNAs.

LncRNA_id	Chromosome	Seq‐length	Fold change	p‐value	Regulation	Gene symbol
NONHSAT186090.1	2	12,147	Positive infinity	0.011	UP	PRKCA
NONHSAT144744.2	17	3567	Positive infinity	0.016	UP	MAVS
NONHSAT167188.1	13	23,214	−9.074	0.018	DOWN	CCL5、MAVS
NONHSAT192274.1	22	28,929	10.056	0.023	UP	CCL5
MSTRG.296268.3	7	366	Positive infinity	0.032	UP	EGFR
NONHSAT193717.1	3	9570	Positive infinity	0.034	UP	CCL5、IRF1
NONHSAT178653.1	18	5353	Positive infinity	0.035	UP	CCL5
NONHSAT151072.1	1	5933	Positive infinity	0.037	UP	CCL5、IRF1
NONHSAT207844.1	6	5996	Positive infinity	0.037	UP	FYN
ENST00000432386	1	4109	Positive infinity	0.04	UP	MAVS
NONHSAT203630.1	5	2899	Positive infinity	0.04	UP	PIK3CD
NONHSAT108196.2	6	14,196	Minus infinity	0.043	DOWN	MAVS、CCL5
NONHSAT144171.2	16	1232	Positive infinity	0.044	UP	LYN
NONHSAT188526.1	20	13,156	Positive infinity	0.044	UP	CCL5、IRF1
NONHSAT200845.1	4	1896	Positive infinity	0.046	UP	CCL5、IRF1
NONHSAT181083.1	2	11,150	Positive infinity	0.047	UP	BECN1
ENST00000513560	5	4769	Positive infinity	0.049	UP	TNFRSF9

### 3.7 Verification and clinical correlation analysis of core immune‐related sEVs‐lncRNAs

**FIGURE 6:**
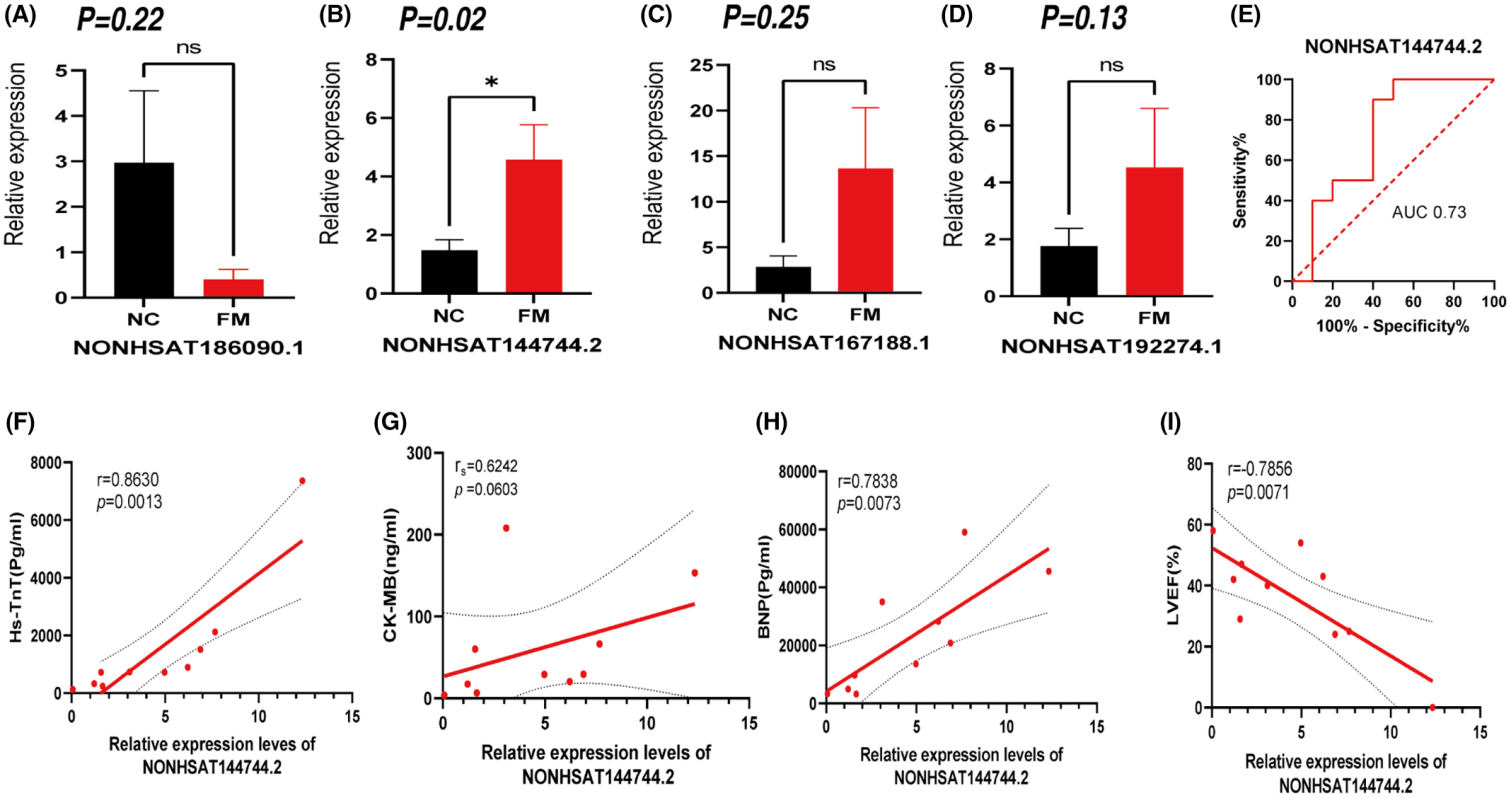
Verification and clinical correlation analysis of selected lncRNA molecules. (A–D) Verification of the four selected lncRNAs assessed by quantitative real time‐PCR (qRT‐PCR). FM: the fulminant myocarditis group; NC: normal control. Data are mean ± standard deviation. (E) The receiver operating characteristic (ROC) curve analysis for NONHSAT144744.2 in the diagnosis of FM. (F–I) Clinical correlation analysis of NONHSAT144744.2 in plasma sEVs.

The top four core immune‐related sEVs‐lncRNAs with *p* values (three increased and one decreased) among the 17 lncRNAs screened in the previous step were selected and their levels were further verified using RT‐qPCR. The results showed that the levels of NONHSAT144744.2 were consistent with the sequencing (*p* < 0.05), while NONHSAT186090.1, NONHSAT167188.1 and NONHSAT192274.1 level changes lost statistical significance (*p* > 0.05) (Figure 6A–D). Previous studies have shown that lncRNAs in plasma sEVs have good potential as diagnostic markers. Therefore, we selected NONHSAT144744.2 as a candidate molecule and plotted its ROC curve (receiver operating characteristic curve) to preliminarily explore its specificity and sensitivity as a diagnostic marker for fulminant myocarditis, as shown in Figure 6E, NONHSAT144744.2 showed good sensitivity and specificity (AUC = 0.73(95% confidence interval [CI], 0.49–0.97)) for the diagnosis of FM in children. We also analysed the correlation between the relative expression of NONHSAT144744.2 and clinical indicators of fulminant myocarditis, such as Hs‐TnT, BNP, CK‐MB and left ventricular ejection fraction (LVEF). The results showed that NONHSAT144744.2 had a good clinical correlation. Its relative expression was positively correlated with Hs‐TnT content (*r* = 0.8630, *p* = 0.0013) and BNP content (*r* = 0.7838, *p* = 0.0073). The correlation with CK‐MB content was not statistically significant (*r*_s_ = 0.6242, *p* = 0.0603), and was negatively correlated with LVEF (*r* = −0.7856, *p* = 0.0071; Figure 6F–I).

## 4 DISCUSSION

FM is the most serious type of myocarditis in children, with an acute onset and severe clinical course.^2^ It has been reported that FM accounts for up to 38% of cases of acute myocarditis in children, and the mortality rate is higher than 48%.^30^ Immune‐related systemic signalling events such as immune response disorders, inflammatory factor storms and circulatory failure caused by direct invasion of pathogenic microorganisms into cardiomyocytes play important roles in the development of FM.^3^ Recent studies have shown that circulating sEVs can perform important functions in the systemic signalling of various diseases such as sepsis^31^ and acute myocardial infarction.^32^ sEVs can carry a variety of signalling molecules, including lncRNA, mRNA, miRNA and proteins which regulate cellular responses to environmental changes or stress signals.^33^, ^34^ Previous studies have shown that the cargo carried by sEVs can also play an important role in immune regulation. For example, Dosil et al. found that miR‐10b‐5p, miR‐92a‐3p and miR‐155‐5p carried by natural killer (NK) cell‐derived EVs promoted GATA3 mRNA down‐regulation and Th1 polarization in CD4^+^ T cells. This is followed by IFN‐γ and IL‐2 production and the induction of MHC‐II and CD86 expression in DCs.^35^

With the increased study of lncRNA, many researchers have found that lncRNA can regulate immune response^36^, ^37^, ^38^, ^39^ and the functions of non‐origin cells via sEVs. For example, Liang Y et al. found that the LncRNA BCRT1, carried by extracellular vesicles derived from breast cancer tissue, can target miR‐1303 to induce the differentiation of macrophages.^40^ Xue Li et al. found that hepatocellular carcinoma ‐derived sEVs‐lncRNA TUC339 can regulate macrophage activation and polarization.^41^ Therefore, characterizing lncRNA in circulating sEVs and analysing their functions could provide new ideas for exploring the occurrence and development of immune‐related diseases.

Previous studies from our group have shown that there are significant differences in lncRNA expression profiles in peripheral blood leukocytes between FM children and healthy control children and that the target genes of differentially expressed lncRNAs are significantly enriched for signalling pathways related to immune regulation.^42^ However, whether the lncRNAs in plasma sEVs differ in children with FM compared to healthy controls and whether the sEVs‐lncRNAs with different levels play a role in immune regulation within FM have not yet been elucidated.

Here, we extracted plasma sEVs from children with FM and healthy children, characterized and compared their morphology, size and concentrations (Figure 1A–D). We then extracted total RNA from the sEVs of five children with FM and five healthy controls for whole‐transcriptome sequencing, as described previously. Seventy‐nine sEVs‐lncRNAs had different levels (fold change >2, *p* < 0.05), 68 lncRNAs had increased levels and 11 had decreased levels. These results indicate that sEVs and sEVs‐lncRNAs are different in the plasma of children with FM.

Then, we predicted the target genes of lncRNAs with different levels and performed functional enrichment analysis, we found that the target genes were mainly enriched for endocytosis, mTOR signalling and protein export signalling pathways. Studies have shown that these pathways play important roles in the pathogenesis of FM.^4^ For example, previous studies have shown that endocytosis plays important roles in signal transduction, mitosis, antigen presentation and cell migration,^43^, ^44^ and it is one of the most important mechanisms through which the monocyte–macrophage system enacts its immunomodulatory role. PI3K/Akt/ mTOR pathway can regulate macrophage polarization by controlling the expression of inflammatory cytokines, phagocytosis, autophagy and cell metabolism.^45^ Protein export is a biological process necessary for important functions of pathogens, including expression of virulence factors on the cell surface, nutrient acquisition, organelle biogenesis and the extracellular release of effector proteins,^46^ playing an important role in the occurrence and development of viral myocarditis. Thus, plasma sEVs‐lncRNAs may regulate the pathogenesis of FM via these signalling pathways.

Researchers have found that abnormal activation of the immune system, excessive polarization and aggregation of macrophages in tissues or organs, and cytokine storms are important factors in the pathogenesis of FM.^23^ In order to further explore the role that differentially expressed sEVs‐lncRNAs may play in the immune adjustment of FM, we screened 75 immune‐related target genes and found that these immune‐related target genes were mainly enriched for antimicrobial and cytokine receptor functions. Enrichment analysis of these immune‐related target genes revealed that they were mainly enriched for GO terms such as positive regulation of cell migration, signal transduction and inflammatory responses. They were also largely involved in cytokine–cytokine receptor interactions and PI3K‐Akt signalling pathways. Cytokine is a kind of low molecular weight soluble protein with a wide range of biological activities. After binding to the corresponding cytokine receptors, it can play a variety of biological functions such as regulating immune response and repairing tissue damage, and play a central role in immune inflammatory disease.^47^ When cytokine storms occur, excessive production of cytokines in the body leads to abnormal activation of the cytokine–cytokine receptor pathway, imbalance between pro‐inflammatory and anti‐inflammatory responses, and severe self‐reinforcement of various feedback mechanisms, eventually leading to systemic tissue damage, multiple organ failure and even death.^48^ Therefore, the regulation of the cytokine–cytokine receptor interaction pathway is essential for regulating the development of cytokine storms during FM. The PI3K‐Akt signalling pathway has been shown to play an important regulatory role in a variety of inflammatory diseases. A recent study has shown that the down‐regulation of lactate dehydrogenase A (LDHA) mediated by the PI3K / Akt‐HIF‐1α pathway can inhibit glycolysis in neutrophils, thereby inhibiting their phagocytosis and chemotaxis, leading to the inhibition of neutrophil immune response.^49^ Therefore, regulating the core genes of PI3K‐Akt signalling pathway can further achieve the purpose of regulating neutrophil immune response. The results of enrichment analysis in our study showed that differentially expressed lncRNAs in plasma sEVs could regulate the expression of related genes in cytokine–cytokine receptor interaction and PI3K‐Akt signalling pathway. Therefore, we speculated that plasma sEVs‐lncRNAs may be involved in the occurrence and development of FM by regulating these immune‐related target genes.

To further understand the interactions between these immune‐related target genes, we constructed a PPI network between these genes using the STRING website. Ten hub genes (EGFR, LYN, FYN, PIK3CD, CCL5, PRKCA, IRF1, MAVS, BECN1 and TNFRSF9) in the network were screened with the cytoHubba plug‐in in Cytoscape 3.9.1. Seventeen core immune‐related sEVs‐lncRNAs were found to regulate these hub genes. After expanding the sample size verification, we found that the expression level of NONHSAT144744.2 was consistent with the sequencing results and the difference was statistically significant, then we conducted a preliminary analysis of the diagnostic potential and clinical relevance of NONHSAT144744.2 in children with fulminant myocarditis, and the results showed that NONHSAT144744.2 has good clinical relevance, diagnostic specificity and sensitivity. We searched the PubMed database and found no functional studies on NONHSAT144744.2. However, we found a study reported that mitochondrial antiviral signalling proteins (MAVS) play an important role in type I interferon‐mediated antiviral responses and are important regulatory proteins in viral myocarditis, especially myocarditis caused by Coxsackie B virus.^50^ MAVS is the target gene of NONHSAT144744.2. Thus, we hypothesized that NONHSAT144744.2 could enter the target cells with sEVs in the plasma during the pathogenesis of myocarditis and regulate the expression of MAVS gene, thus regulating the antiviral response of target cells.

In summary, this study is the first to extract and validate sEVs from the plasma of children with FM, provided a comprehensive characteristic analysis of plasma sEVs‐lncRNAs in this population, and screened lncRNAs molecules that play a central role in immune regulation. We also used GO and KEGG pathway enrichment analysis, PPI network construction to explore the role of sEVs‐lncRNAs with different levels in the development of FM and to explore possible immunomodulatory mechanisms. Taken together, our results provide a new theoretical basis for further study of the role of sEVs and lncRNAs in FM.

Our study has some limitations. First, because of the low incidence of FM and the lack of available myocardial biopsies, we had a small sample size, and further multicentre studies with larger samples should be performed, moreover, due to the small sample size, the results obtained in the process of verifying sequencing data were statistically insignificant Second, sEVs can be lost during storage, and it is easy to get impurities (including apolipoproteins) in sEVs extracted with differential centrifugation, this results in low purity and concentrations of RNA in extracted sEVs, meaning that it can be difficult to actually detect the quantity of RNA, and the difficulty of RNA quantification will lead to incorrect results in the later verification process Thus, it is important to improve the enrichment methods used to study sEVs, reduce losses, and improve the RNA extraction steps in order to obtain higher purity and concentrations of RNA. Third, this study only predicted the possible mechanisms of lncRNA through database and enrichment analysis, and further experimental verification is needed to clarify these specific mechanisms.

## AUTHOR CONTRIBUTIONS

**Mengjie Ma:** Data curation (lead); formal analysis (lead); writing – original draft (lead); writing – review and editing (equal). **Siyu Chen:** Investigation (equal); methodology (equal). **Xinyue Zhang:** Investigation (equal); methodology (equal). **Rulin Yang:** Supervision (equal); validation (equal); writing – review and editing (equal). **Li Zhang:** Supervision (lead); validation (equal); writing – review and editing (equal). **Kaiyin Guo:** Supervision (equal); validation (equal); writing – review and editing (equal). **Jing Wang:** Conceptualization (lead); visualization (equal). **Hailin Jia:** Conceptualization (supporting); visualization (equal). **Yingnan You:** Data curation (lead). **Bo Han:** Conceptualization (lead); funding acquisition (lead); resources (lead).

## FUNDING INFORMATION

This study was funded by the National Natural Science Foundation of China (no. 81873498); Jinan Science and Technology Development Plan (no. 202134015); Special Expert of Taishan Scholars (no. ts201511099); the Natural Science Foundation of Shandong Province, China (ZR2023MH181, ZR2023QH296).

## CONFLICT OF INTEREST STATEMENT

These authors have no conflict of interest.

## Supporting information


Tables S1‐S3.

## Data Availability

The datasets (GENERATED) for this study can be found in the (GSE226412; https://www.ncbi.nlm.nih.gov/geo/query/acc.cgi?acc=GSE226412).
