# Supplementary material for: Identification and functional analysis of circulating small extracellular vesicle lncRNA signatures in children with fulminant myocarditis
Source: J Cell Mol Med. 2023 Nov 9;28(2):e18034. doi: 10.1111/jcmm.18034 (PMC10826448; doi:10.1111/jcmm.18034)
Supplement: Supplementary file 1 — Tables S1‐S3. [file JCMM-28-e18034-s001.docx]

**Supplementary Table 1**. Specific primers for quantitative real time-PCR.

| **Primer name** | **Primer sequence (5’-3’)** |
| --- | --- |
| NONHSAT186090.1 | F:5’ACCAGGATGACAACCCTTCAC3’  R:5’TGGTCCTGCTCTGGATGTTC3’ |
| NONHSAT144744.2 | CGCCAGTGTTTCCAGAGAGT  TGGTTCCCACCACTCAAAGG |
| NONHSAT167188.1 | F:5’CAGCCACCCAAAGTCTACCA3’  R:5’AAGACTGAGAACCACCCTGC3’ |
| NONHSAT192274.1 | **F:5’** CTAGGGGTTTCTTGGGGCTT3’  R:5’GGTAGGTTGGGTCTTGCTCC3’ |
| GAPDH | F:5’CAAGAAGGTAGTGAAGCAGGC3’ R:5’TGTTGAAGTCGGA GGAGACC3’ |

F: Forward; R: Reverse.

**Supplementary Table 2.** Detailed information about the fulminant myocarditis (MF) and normal control (NC) groups.

| **Sample ID** | **Gender** | **Age(years)** | **Hs-TnT(Pg/ml)** | **BNP(Pg/ml)** | **CK-MB(ng/ml)** | **LVEF(%)** |
| --- | --- | --- | --- | --- | --- | --- |
| FM01 | Female | 11 | 4011.00 | 7155.00 | 11.24 | 35 |
| FM02 | Female | 8 | 1518.00 | 12329.00 | 40.24 | 45 |
| FM03 | Male | 4 | 3816.00 | 5716.00 | 300.00 | 40 |
| FM04 | Male | 13 | 990.00 | 4318.00 | 39.40 | 53 |
| FM05 | Female | 16 | 8007.00 | 13335.00 | 156.60 | 18 |
| FM06 | Female | 8 | 244.30 | 3235.00 | 6.60 | 47 |
| FM07 | Female | 11 | 724.70 | 13625.00 | 29.02 | 54 |
| FM08 | Female | 12 | 2122.00 | 59041.00 | 66.30 | 25 |
| FM09 | Female | 5 | 897.50 | 28332.00 | 20.38 | 43 |
| FM10 | Female | 10 | 329.00 | 4942.00 | 17.34 | 42 |
| FM11 | Male | 9 | 7361.00 | 45583.00 | 153.40 | 0* |
| FM12 | Male | 6 | 1515.00 | 20812.00 | 29.30 | 24 |
| FM13 | Female | 11 | 127.80 | 3363.00 | 3.79 | 58 |
| FM14 | Male | 12 | 724.70 | 9747.00 | 60.10 | 29 |
| FM15 | Male | 3 | 737.00 | 35000.00 | 208.00 | 40 |
| NC01 | Male | 11 | Normal | Normal | Normal | 65 |
| NC02 | Female | 13 | Normal | Normal | Normal | 63 |
| NC03 | Female | 8 | Normal | Normal | Normal | 65 |
| NC04 | Male | 4 | Normal | Normal | Normal | 64 |
| NC05 | Female | 9 | Normal | Normal | Normal | 64 |
| NC06 | Female | 15 | Normal | Normal | Normal | 63 |
| NC07 | Male | 4 | Normal | Normal | Normal | 65 |
| NC08 | Female | 11 | Normal | Normal | Normal | 63 |
| NC09 | Male | 8 | Normal | Normal | Normal | 65 |
| NC10 | Female | 12 | Normal | Normal | Normal | 65 |
| NC11 | Male | 13 | Normal | Normal | Normal | 63 |
| NC12 | Male | 16 | Normal | Normal | Normal | 64 |
| NC13 | Female | 12 | Normal | Normal | Normal | 64 |
| NC14 | Male | 6 | Normal | Normal | Normal | 63 |
| NC15 | Male | 13 | Normal | Normal | Normal | 65 |

BNP: Brain natriuretic peptide, an index of heart failure (normal range, 0–450 pg/ml); CK-MB: creatine kinase isoenzymes-MB, an indicator of myocardial injury (normal range, 0–5ng/ml); Hs-TnT: Hypersensitive troponin T, a myocardial injury marker (normal range, 3–14pg/ml); LVEF: Left ventricular ejection fraction, tested by echocardiography, indicating cardiac contraction ability (normal value ＞60%). 0^*^: Myocardial motion disappeared completely.

**Supplementary Table3.** Clinical presentations of fulminant myocarditis patients.

| **Sample** | **Symptoms** | **BP (mmHg)** | **ECG** | **Phenotypes** | **MCS** | **Follow-up** |
| --- | --- | --- | --- | --- | --- | --- |
| FM01 | Abdominal distension, fatigue, chest tightness, cough | 84/47 | ST-T change | Acute heart failure | ECMO | Recovery |
| FM02 | Fever, abdominal pain | 79/42 | III^◦^ AVB | Adams-Strokes syndrome,  Cardiac shock | TP | Recovery |
| FM03 | Fever, emesis, abdominal discomfort | 104/90 | VT | Acute heart failure,  Cardiac shock | None | Recovery |
| FM04 | Chest pain | 114/63 | Sinusbradycardia  , ST-T change | Acute heart failure, | None | Recovery |
| FM05 | Fatigue, emesis,  abdominal discomfort | 83/58 | III^◦^ AVB | Cardiac shock | TP/ECMO | Death |
| FM06 | Fever, cough, abdominal pain | 98/57 | ST-T change | Acute heart failure,  Cardiac shock | None | Recovery |
| FM07 | Fever, convulsion | 97/70 | ST-T change | Acute heart failure | None | Recovery |
| FM08 | Fever, weak, cyanosis | 47/35 | VF, VT | Cardiac shock | ECMO | Death |
| FM09 | Emesis, abdominal pain  syncope | 65/37 | Inverted T wave | Cardiac shock,  Adams-Strokes syndrome,  Acute heart failure | None | Recovery |
| FM10 | chest distress, fatigue, headache, chest pain | 90/69 | Inverted T wave | Cardiac shock,  Adams-Strokes syndrome,  Acute heart failure | ECMO | Recovery |
| FM11 | Diarrhea, fever, fatigue | 85/50 | III^◦^ AVB | Acute heart failure,  Cardiac shock | TP | Death |
| FM12 | Fever, emesis, abdominal pain, headache | 101/65 | Inverted T wave  PAC, PVC, VT | Acute heart failure,  Cardiac shock | ECMO | Recovery |
| FM13 | Syncope, convulsion | 133/59 | III^◦^ AVB | Acute heart failure,  Cardiac shock | TP | Recovery |
| FM14 | Nausea, emesis | 85/51 | Inverted T wave  VT | Acute heart failure,  Cardiac shock | ECMO | Recovery |
| FM15 | Fever, cough, emesis | 85/52 | ST-T change | Cardiac shock | None | Recovery |

AVB: Atrioventricular block; BP: Blood pressure at admission; ECG: Electrocardiograph; ECMO: Extracorporeal membrane oxygenation; MCS: mechanical circulatory support; TP: Temporary pacemaker; VF: Ventricular fibrillation; VT: Ventricular tachycardia.
